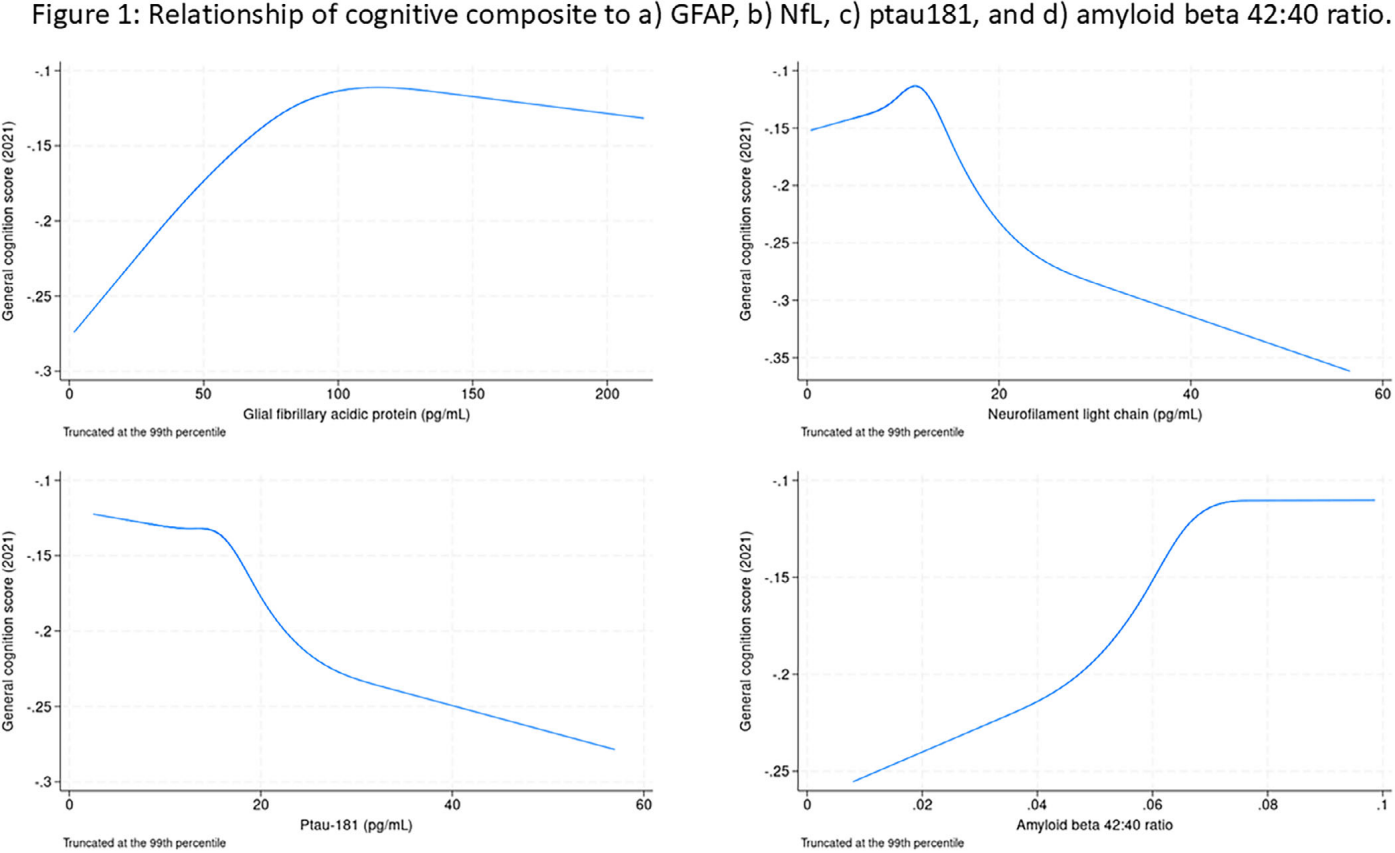# Association of Blood‐based Biomarkers of Alzheimer's Disease with Cognition and Educational Experiences in Midlife

**DOI:** 10.1002/alz70860_107507

**Published:** 2025-12-23

**Authors:** Jennifer J. Manly, Adam Brickman, Soobin Kim, Michael Culbertson, Eric Grodsky, Chandra Muller, John Robert Warren

**Affiliations:** ^1^ Department of Neurology, Gertrude H. Sergievsky Center and the Taub Institute for Research on Alzheimer's Disease and the Aging Brain, New York, NY, USA; ^2^ University of Wisconsin ‐ Madison, Madison, WI, USA; ^3^ University of Texas at Austin, Austin, TX, USA; ^4^ University of Minnesota, Minneapolis, MN, USA

## Abstract

**Background:**

Development of blood‐based biomarkers makes measurement of neurodegeneration possible in populations that have not traditionally been represented in ADRD research. Investigation of the relationship of blood‐based AD biomarkers to cognitive functioning in longitudinal population representative samples is critical. This study examined the relationship between plasma AD biomarkers and cognitive functioning in middle‐aged adults, and determined whether educational experiences collected in 1980 while they were in high school (including school contexts, high school academic performance, and educational attainment) moderate this relationship. We also investigated whether these associations differed by early life SES, sex/gender, race and ethnicity, and US region.

**Method:**

High School and Beyond (HS&B:80) assessed a nationally representative cohort of people who were in high school in 1980, and 41 years later, collected telephone‐ and web‐based measures of word list learning and memory, paired associates learning, fluency, and number span at age ∼60 years. A measure of global cognitive functioning was calculated using IRT. Amyloid beta 42:40 ratio, pTau‐181, NfL, and GFAP levels were assayed from plasma. Regression models examined linear and nonlinear associations between cognitive measures and plasma biomarkers.

**Result:**

Participants (*n* = 4,340) ranged in age from 56 to 63 (mean = 58), were 55% women, 60% were White, 21% were Latinx, and 14% were Black. Better global cognition was associated with lower plasma *p*‐tau‐181 (β = ‐.003, 95% CI ‐0.110 to ‐0.015) and NfL (β = ‐.004, 95% CI ‐0.006 to ‐0.002), and with higher AB 40/42 ratio (β = 1.388, 95% 0.207 to 2.568). Higher GFAP was associated with better cognition (β = .001, 95% CI 0.000 to 0.001). Among the individual measures, paired associate learning was most consistently associated with plasma biomarkers. There was no effect modification by educational experience, early life SES, race and ethnicity, sex/gender, or region of the US.

**Conclusion:**

At age 60 in a population representative sample, blood based biomarkers account for almost none of the observed variation in cognitive function, and these relationships do not vary by early life SES, race and ethnicity, sex/gender, US region, or educational experience.